# Selective Constraint on the Upstream Open Reading Frames That Overlap with Coding Sequences in Animals

**DOI:** 10.1371/journal.pone.0048413

**Published:** 2012-11-01

**Authors:** Ming-Kung Hsu, Feng-Chi Chen

**Affiliations:** 1 Division of Biostatistics and Bioinformatics, Institute of Population Health Sciences, National Health Research Institutes, Zhunan, Miaoli County, Taiwan; 2 Department of Life Sciences, National Chiao-Tung University, Hsinchu, Taiwan; 3 Department of Dentistry, China Medical University, Taichung, Taiwan; J. Craig Venter Institute, United States of America

## Abstract

Upstream open reading frames (uORFs) are translational regulatory elements located in 5′ untranslated regions. They can significantly repress the translation of the downstream coding sequences (CDS), and participate in the spatio-temporal regulations of protein translation. Notwithstanding this biological significance, the selective constraint on uORFs remains underexplored. Particularly, the uORFs that partially overlap with CDS with a different reading frame (overlapping uORFs, or “VuORFs”) may lead to strong translational inhibition or N-terminal truncation of the peptides encoded by the affected CDS. By analyzing VuORF-containing transcripts (designated as “VuORF transcripts”) in human, mouse, and fruit fly, we demonstrate that VuORFs are in general slightly deleterious - the proportion of genes that encode at least one VuORF transcript is significantly smaller than expected in all of the three examined species. In addition, this proportion is significantly smaller in fruit fly than in mammals, indicating a higher efficiency of removing VuORFs in the former species because of its larger effective population size. Furthermore, the deleterious effect of a VuORF depends on the sequence context of its start codon (VuAUG). VuORFs with an optimal VuAUG context are more strongly disfavored than those with a suboptimal context in all of the three examined species. And the propensity to remove optimal-context VuAUGs is stronger in fruit fly than in mammals. Intriguingly, however, the currently observable optimal-context VuAUGs (but not suboptimal-context VuAUGs) are more conserved than expected. These observations suggest that the regulatory functions of VuORFs may have been gained fortuitously in organisms with a small effective population size because the slightly deleterious effect of these elements can be better tolerated in these organisms, thus allowing opportunities for the development of novel biological functions. Nevertheless, once the functions of VuORFs were established, they became subject to negative selection.

## Introduction

Upstream open reading frames (uORFs) are open reading frames that are located in 5′ untranslated regions (5′UTRs). A uORF contains one upstream start codon (uAUG), one in-frame stop codon, and at least one non-stop codon in between. These regulatory elements are known to play an important role in translational regulations [1], [2], [3], [4], [5]. Particularly, uORFs can significantly repress the translation of their downstream coding sequences [1], [2], [6], [7], [8]. They are also known to be involved in a number of human diseases [6], [7]. Given their functional importance, uORFs have been reported to be the target of natural selection [1], [9], [10].

uORFs can be classified into different types according to their positions and reading frames relative to the downstream coding sequence (CDS) [9]. The most common type of uORF is strict uORF (designated as “SuORF”), which is included entirely within a 5′UTR. A second type of uORF – the overlapping uORF (designated as “VuORF”) – extends beyond 5′UTR and partially overlaps with the main CDS with a different reading frame. Both SuORFs and VuORFs can “hijack” the translational machinery, leading to strong inhibition of translation of the downstream CDS (quantitative change). Interestingly, VuORFs have an addition effect – it can cause skipping of the annotated translational start codon (sAUG), and result in an N-truncated peptide (qualitative change) when translation restarts at a downstream AUG triplet [11]. Furthermore, VuORFs are suggested to have a stronger translational inhibitory effect because they have a higher probability of causing sAUG skipping than SuORFs [9]. VuORFs were reported to participate in the regulations of condition-specific protein expressions [12], [13], [14], [15]. However, the number of experimentally validated functional VuORFs is fairly limited. The biological and evolutionary significance of VuORFs thus remains unclear. It is unknown whether most of the VuORFs are biologically functional, selectively neutral, or deleterious. Considering the repressive effects of VuORFs on protein expression level, which is highly conserved across species [16], [17], we hypothesize that most of the VuORFs are deleterious. Furthermore, since VuORFs affect only specific transcripts (rather than all of the transcript isoforms of a gene), the fitness effects resulting from the occurrences of these elements may be relatively small and can be better observed by comparing organisms with different effective population sizes (N_e_), for organisms with a larger N_e_ can more efficiently remove slightly deleterious genetic elements than those with a smaller N_e_.

In this study, we examined the abovementioned hypothesis from several different angles. Firstly, we conducted a simulation study by randomly reshuffling the 5′UTR sequences of the transcripts (so that the G+C contents and the lengths of 5′UTRs can be controlled) in three model organisms: human (*Homo sapiens*), mouse (*Mus musculus*), and fruit fly (*Drosophila melanogaster*). If VuORFs have been deleterious, the actual proportion of genes encoding VuORF-containing transcripts (or “VuORF transcripts” in short) should be smaller than that derived from the random data. Otherwise the two proportions should be approximately equal to each other.

Secondly, we compared the proportions of genes that encode VuORF transcripts in the three studied model organisms, which have an N_e_ of approximately 10^4^ (human), 10^5^ (mouse), and 10^6^ (fruit fly), respectively [18], [19], [20]. If VuORFs are slightly deleterious, fruit fly should have the smallest proportion of genes that encode VuORF transcripts, and human should have the largest among the three species, for the efficiency of natural selection is positively correlated with N_e_. This expected difference in the proportion of genes encoding VuORF transcripts should be unobservable if most VuORFs are biologically functional or selectively neutral.

Thirdly, we examined the sequence contexts of uAUGs and sAUGs in the VuORF transcripts. Previous studies have shown that sAUGs tend to have an optimal sequence context to facilitate efficient translation of the main CDS [21], [22]. We reason that selection should have favored a non-optimal context for the uAUGs of VuORFs (termed “VuAUGs”) because an optimal-context VuAUG will potentially aggravate the deleterious effects of the VuORF, leading to a remarkable reduction in fitness and rapid removal of the VuORF. Such a preference for non-optimal VuAUG context should be stronger in fruit fly than in mammals, again because of the differences in the efficiency of natural selection.

Finally, we examined the cross-species conservation of VuAUGs that are currently observable in the genomes of the three examined species. Note that if VuORFs have been deleterious, the majority of the ancestral VuORFs (and thus the VuAUGs) should have been eliminated by natural selection. Therefore, the currently observable VuORFs either have emerged very recently (so that they have not been eliminated by selection), or they are biologically functional (so that they are subject to negative selection and tend to remain in the genome). In the latter case, the VuAUGs are expected to be highly conserved across multiple genomes. In the former case, however, the VuAUGs should occur only in the genomes of very closely related species.

Our results indicate that the proportion of genes that encode VuORF transcript(s) is significantly smaller than expected for all of the three analyzed species. Furthermore, among the three analyzed species, human has the largest and fruit fly has the smallest proportion of genes encoding VuORF transcript(s). We also found that VuORFs with an optimal-context VuAUG are more strongly disfavored than those with a suboptimal-context VuAUG. Interestingly, however, the currently observable optimal-context VuAUGs are more conserved than expected. Taken together, these results suggest that although most of the ancestral VuORFs might be deleterious, the currently observable VuORFs (particularly those with an optimal-context VuAUG) are perhaps biologically functional. Our study may thus bring new insights into the evolution of these translational regulatory elements in complex organisms.

## Results and Discussion

### Overlapping uORFs are in General Slightly Deleterious

We first retrieved all of the known transcripts that have known protein products for human, mouse, and fruit fly via the UCSC Genome Browser (see Materials and Methods for more details). We then randomly shuffled the 5′UTRs of these transcripts for 1,000 times. For each reshuffling experiment, we examined whether a VuORF was observable in the reshuffled transcripts, and calculated the proportion of genes that contained at least one VuORF transcript. We thus obtained a theoretical distribution of the proportion of genes containing VuORF transcripts given the lengths, G+C contents, and coding sequences of the current transcripts for each of the three examined species. If the actual proportion of VuORF transcript-encoding genes deviates significantly from this theoretical distribution, we may infer that VuORFs are subject to certain selection pressures. Note that coding sequences are not reshuffled here because they are usually highly conserved. For comparison, we also conducted the same analysis for SuORFs.

**Figure 1:**
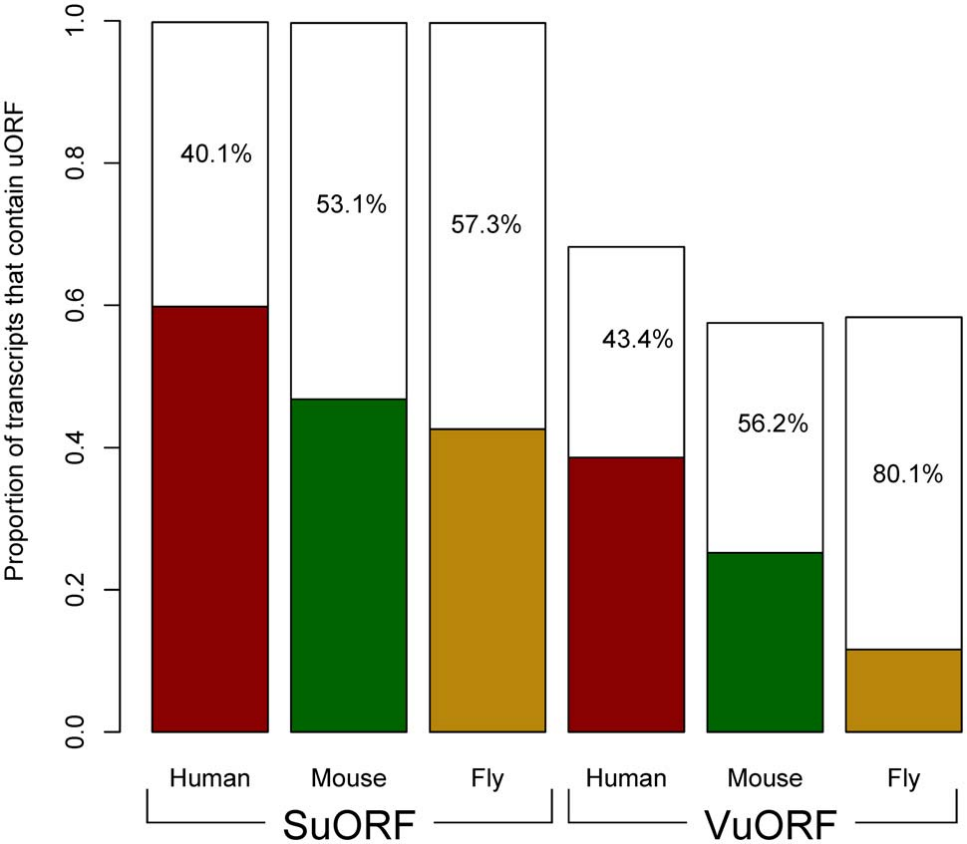
The theoretical (colored + blank bars) and observed (the colored bars) proportions of genes that encode at least one SuORF (left half) or VuORF transcript (right half) in human, mouse, and fruit fly. The numbers indicate the percentages of uORFs that potentially have been eliminated by natural selection (the length of the blank bar divided by the full length of the bar). Note that all of the pair-wise differences in the observed proportion (the colored part) are statistically significant either for the same type of uORF (*e.g.*, for SuORF human > mouse > fly; by Chi-square test) or for the same species (*e.g.*, for human, SuORF > VuORF; by t-test). All *p* values < E−15. Also note that the 95% confidence intervals are not shown because of the very narrow ranges.

This simulation study yields several interesting results. Firstly, as shown in Figure 1, for all of the three examined species, the actual percentages of genes that encode at least one uORF transcript are far below the expected values for both SuORFs and VuORFs. For SuORFs, the observed percentages are 59.8%, 46.8%, and 42.6%, respectively, for human, mouse, and fruit fly, as compared with the theoretical 95% confidence interval of 99.8∼99.9%, 99.7∼99.9%, 99.7∼99.9%. Meanwhile, for VuORFs, the observed (theoretical) percentages are 38.6% (68.2∼69.5%), 25.2% (57.5∼59.1%), and 11.6% (58.3∼60.4%), respectively, for human, mouse, and fruit fly. This observation implies that a significant proportion of both SuORFs and VuORFs have been eliminated by natural selection, suggesting that these regulatory elements are likely deleterious.

Secondly, for both SuORFs and VuORFs, the observed percentage of genes containing uORF transcripts decreases with effective population size (*i.e.*, human > mouse > fly; all pair-wise differences are statistically significant, *p*<1.1 E−60, by Chi-square test; Figure 1). This observation is consistent with our prediction that VuORFs should be more effectively removed in organisms with a large N_e_. Interestingly, the same comment appears also applicable to SuORFs. One potential caveat in this analysis is that the number of uORFs may increase with the length of 5′UTR. Of note, fruit fly has been reported to have longer 5′UTRs than mouse [23], [24]. Therefore, the larger number of VuORFs in mouse than in fly is unlikely to result from the difference in 5′UTR length. Nevertheless, we still controlled for 5′UTR length and conducted the study again for VuORFs. As shown in Figure S1, the negative correlation between N_e_ and the percentage of VuORF transcript-containing genes holds well when the length of 5′UTR is controlled. Therefore, our result does not seem to be affected by the difference in 5′UTR length between species.

Thirdly, the percentages of uORFs potentially eliminated by selection (the blank bar) are significantly larger for VuORFs than for SuORFs regardless of species (human: 43.4% Vs. 40.1%; mouse: 56.1% Vs. 53.1%; fly: 80.1% Vs. 57.3%; all *p* values < E−15 by t-test; Figure 1). These percentages were calculated by (1- observed percentage/theoretical percentage). For example, for human SuORFs, the percentage of eliminated SuORFs is (1–59.8/99.8), where 99.8 is the lower bound of the theoretical 95% confidence interval. This result further supports our hypothesis that VuORFs are in general slightly deleterious, so that they are more efficiently removed in fruit fly than in mouse and human. In addition, VuORFs appear to be more deleterious than SuORFs, so that a larger proportion of VuORFs have been eliminated, especially in the species with the largest N_e_ (fly). Note that if VuORFs are strongly deleterious, we could not have observed such differences because the vast majority of these uORFs would have been eliminated, and the proportions of currently observable VuORF transcript-encoding genes should have been close to zero in all of the three species.

### The Selective Constraint on VuORFs is Associated with the Sequence Contexts of sAUGs and VuAUGs

Next, we examined whether the selection pressure imposed on VuORFs are associated with the sequence context of sAUGs. To address this issue, we used only the transcripts that include a single VuORF and no other types of uORFs. We term these transcripts “single-VuORF transcripts”. This filtering procedure is necessary because according to the scanning model of protein translation [25], premature translation may occur at uORFs other than VuORFs if the former are closer to the 5′cap of the transcript. In such cases, the sequence context of the VuAUG may be unimportant because the translational machinery has been “hijacked” by the upstream uORF. After the filtering process, we obtained 2,951, 2,056, and 580 single-VuORF transcripts, respectively, for human, mouse, and fruit fly (Table 1). As expected, for all of the three analyzed species, the transcripts with an optimal-context sAUG far outnumber those with a suboptimal-context sAUG in all of the three examined species (Table 1), which is consistent with previous findings [26]. The significantly larger proportion of optimal-context sAUGs than suboptimal-context sAUGs is due to the requirement for efficient translation of the main CDS [26]. Interestingly, the proportion of single-VuORF transcripts that have an optimal sAUG context actually increases with N_e_ – fruit fly has the largest proportion (79.3%), followed by mouse (77.2%), and lastly by human (73.2%) (the fly-human and mouse-human differences are both statistically significant; *p*<0.01 by Chi-square test). And of course, the proportions of suboptimal sAUG context follow the reverse order (Table 1; both fly-human and fly-mouse differences are statistically significant; *p*<0.01 by Chi-square test). For comparison, we also calculated the genome-wide average of optimal and suboptimal sAUG usage by randomly selecting one transcript for each protein coding gene. Interestingly, as shown in Table 1, the percentage of optimal sAUGs in single-VuORF transcripts is larger than the genome-wide average regardless of species (all *p* values < E−16 by Chi-square test), whereas the opposite is true for suboptimal sAUGs. This observation suggests that the incorporation of a VuORF in a transcript is related to the increased usage of optimal sAUGs. Note that the difference in the proportion of optimal uAUG-context VuORFs between species is unrelated to the difference in G+C content in 5′UTRs. This is because an optimal context is defined as a purine (A or G) at the -3 position relative to the AUG triplet, whereas a suboptimal context is defined as a pyrimidine (C or T) at the same position (Materials and Methods).

**Table 1:** The numbers (percentages) of single-VuORF transcripts that have an optimal-context (or suboptimal-context) sAUG in human, mouse, and fruit fly as compared with the genome-wide average.

		No. of transcripts	No. (percent) of transcriptswith an optimal-contextsAUG	No. (percent) of transcripts withan suboptimal-contextsAUG
Human	Single VuORF transcripts	2,951	2,159 (73.2%)	792 (26.8%)
	Genome-wide	21,018	10,723 (51.0%)	1,0295 (49.0%)
Mouse	Single VuORF transcripts	2,056	1,587 (77.2%)	469 (22.8%)
	Genome-wide	21,111	11,222 (53.2%)	9,889 (46.8%)
Fly	Single VuORF transcripts	580	460 (79.3%)	120 (20.7%)
	Genome-wide	14,869	9,396 (63.2%)	5,473 (36.8%)

**Figure 2:**
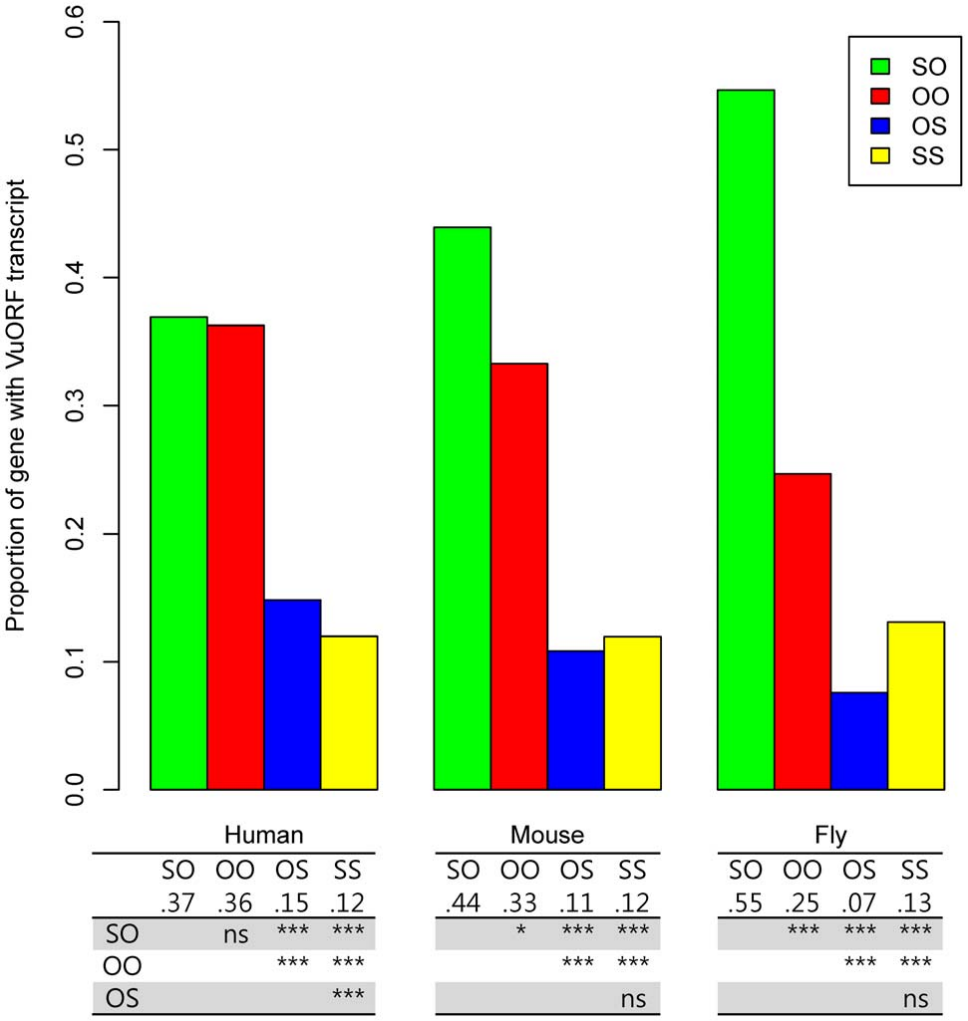
The proportions of different types VuORF transcripts in human, mouse, and fruit fly. The table at the bottom shows the statistical significance (by Chi-square test) in pair-wise comparisons between different types of VuORF transcripts. Note that the sum of proportions may be slightly larger than one because of round-ups. OO: optimal-optimal; OS: optimal-suboptimal; SO: suboptimal-optimal; SS: suboptimal-suboptimal. Statistical significance: *: *p*<0.05; **: *p*<0.01; ***: *p*<0.001; ns: not significant.

We then took the context of VuAUG also into consideration and classified single-VuORF transcripts into four groups (OO, SO, OS, and SS – with the first and second letter representing the context type of VuAUG and sAUG, respectively). As expected, the suboptimal-optimal (SO) type accounts for the largest fraction of single-VuORF transcripts in all of the three analyzed species. Furthermore, the proportion of the SO-type transcripts increases with N_e_ (Human: 36.9%; Mouse: 43.9%; Fly: 54.7%; both human-mouse and mouse-fly differences are statistically significant; *p*<0.001 by Chi-square test) (Figure 2). By contrast, the proportions of the optimal-suboptimal (OS) type of single-VuORF transcripts decreases with N_e_ (Human: 14.8%; Mouse: 10.8%; Fly: 7.6%; both human-mouse and mouse-fly differences are statistically significant; *p*<0.01 by Chi-square test) (Figure 2). The proportions of the optimal-optimal type (OO) also follow the same order as that of the OS-type transcripts. Notably, the proportions of OO-type transcripts are more than twice those of the OS-type transcripts (Figure 2), possibly because an optimal sAUG context is strongly favored by selection over a suboptimal one in all of the three species. Furthermore, the proportion of suboptimal VuAUG context (SO+SS) differs from that of optimal VuAUG context (OO+OS) by a much larger magnitude in fruit fly (67.8% Vs. 32.2%) than in mouse (55.9% Vs. 44.1%) and human (48.9% Vs. 51.1%) (Figure 2). Notably, the difference between the percentages of optimal context- and suboptimal context-VuAUGs actually increases with N_e_ (−2.2% in human, 11.8% in mouse, and 35.5% in fruit fly).

Note that the SO-type and OS-type of transcripts represent two extremes. The SO type is expected to be favored because in this case, the translational machinery may skip the suboptimal VuAUG context, and bind to the sAUG with high efficiency to initiate translation of the main coding sequence. By contrast, the OS type of context may cause strong inhibition of translation because in this case, the VuAUG can bind the translational machinery with high affinity, and the sAUG tends to be skipped. The strong preference for SO-type over OS-type transcripts in fruit fly (as compared with those in mammals) suggests that the selection pressure on VuORFs is associated with the sequence contexts of VuAUGs and sAUGs, and that the strength of such selection pressure is positively correlated with N_e_. This is perhaps because an optimal VuAUG context may aggravate the deleterious effect of the VuORF, and increase the probability of its removal by natural selection. This notion is also supported by the larger difference between the proportion of suboptimal-context VuAUG and that of optimal-context VuAUG in fruit fly than in mammals.

One potential caveat in the above analyses is that we used all of the available genes rather than just orthologous genes for comparisons among the three species. This raises a concern that the above observations may have reflected functional biases or the effects of other lineage-specific factors. To address this issue, we retrieved human-mouse-fruit fly one-to-one orthologous genes and conducted the analyses again. We actually obtained similar results (Figure S2). Meanwhile, since the annotations of transcript isoforms may differ between databases, we also used an alternative dataset (known transcripts from the Ensembl database) for the same analyses and again obtained similar results. (Figure S3).

Another potential caveat is that the higher percent of VuORF transcript-encoding genes in mammals than in fruit fly may be ascribed to differences in study intensity. That is, mammalian genes may include more annotated transcript isoforms (and thus more VuORF transcripts) because they have been more intensively studied than the genes of fruit fly. To examine whether this is true, we analyzed the average numbers of supporting ESTs and cDNAs per analyzed gene (an indicator of study intensity) for the three species. According to the UCSC data, human genes indeed have the largest average number of supporting ESTs/cDNAs and fruit fly genes have the smallest (Figure S4, left panel). However, we obtained the contrary result (fly > mouse > human) from the Ensembl data (Figure S4, right panel). Since both of the UCSC and Ensembl datasets yield similar results that fruit fly has the largest proportion of VuORF-containing genes, biases in study intensity may not fully explain our observations.

### The Cross-species Conservation of Overlapping uORFs

We have shown that most of the ancient VuORFs are perhaps deleterious and have been eliminated by natural selection. However, it is likely that some of the currently observable VuORFs are biologically functional, so that they remain in the current genomes. To examine whether this is true, we analyzed the level of cross-species conservation of the VuAUGs in the human, mouse, and fruit fly single-VuORF transcripts because a VuAUG is an indispensable part of a VuORF. For simplicity, we define the level of VuAUG conservation as the proportion of mammalian genomes (or *Drosophila* genomes) that have an AUG triplet at exactly the same genomic position as the VuAUG of interest according to the UCSC multiple genome alignments (see Materials and Methods for more details). We reason that if a currently observable VuORF is functionally important, at least its VuAUG should be conserved across multiple species. And the level of conservation may reflect the importance of these potential regulatory elements.

**Figure 3:**
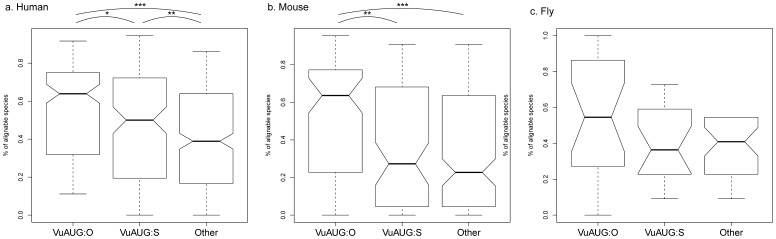
The levels of conservation of VuAUGs and other triplets composed of A, U, and G (AGU, UAG, UGA, GAU, and GUA). “uAUG O”: optimal-context VuAUG; “uAUG S”: suboptimal-context VuAUG; “Other”: other triplets. Statistical significance (by Wilcoxon Rank Sum test): *: *p*<0.05; **: *p*<0.01; ***: *p*<0.001.

Interestingly, as shown in Figure 3, optimal-context VuAUGs are significantly more conserved than suboptimal-context VuAUGs and other triplets composed of A, U, and G nucleotides (i.e. AGU, UAG, UGA, GAU, and GUA) in human and mouse (*p*<0.001 by Wilcoxon Rank Sum test) but not in fly (Figure 3). This finding suggests that the surviving mammalian VuAUGs, particularly those with an optimal context, may have biological functions. Indeed, previous studies [1], [2], [3] have indicated that some VuORFs play an important role in spatio-temporal regulations of protein translation. Notably, the numbers of VuORFs with an optimal-context VuAUG are actually smaller than those with a suboptimal-context uAUG in the current human, mouse, and fruit fly genomes. This appears to suggest that an optimal VuAUG may be favored in currently functional VuORFs, although it is selectively disfavored in view of longer-term evolution.

### Analysis of Single Nucleotide Polymorphisms at VuAUGs

An alternative way to evaluate the selective constraint on VuORFs is to compare the level of polymorphism and the level of divergence at VuAUGs. If currently observable VuORFs are functionally important, they should be subject to negative selection, leading to both a high level of cross-species conservation and a low level of polymorphism. Meanwhile, if VuORFs are slightly deleterious, they should be subject to relaxed negative selection, particularly in human because of the reduced N_e_. In this case, the level of polymorphism at VuAUGs should have been increased. Since polymorphism data are most abundant for human, we only examined the single nucleotide polymorphisms (SNPs) for human VuAUGs by referring to HapMap Phase III data (release #28; http://hapmap.ncbi.nlm.nih.gov/) [27], and compared the human SNPs with the chimpanzee genomic sequence in the UCSC multiple sequence alignment to infer the ancestral state. However, only 18 out of the 2,951 examined VuAUGs were found to contain SNPs (Table S1). We also examined the level of polymorphisms of VuAUGs by referring to the One-thousand Genome Pilot One data. However, we did not find any SNPs for the examined VuAUGs. Although this lack of polymorphism at VuAUGs appears to support the functional importance of the currently observable VuORFs, caution must be taken because the low level of polymorphism may have resulted from the incompleteness of the HapMap data. Furthermore, given the small number of SNPs, it is difficult to make statistically meaningful inferences. More evidence is required to determine whether the polymorphism level at VuAUGs is indeed lower than expected.

### The Evolution of Overlapping uORFs – a Neutral Hypothesis

One important question here is why VuORFs are slightly deleterious. A possible reason is that a VuORF may occur in only one or a few (but not all) of the transcript isoforms of a gene, thus affecting only a limited amount of proteins. Therefore, the fitness effects of these elements are limited. Another possible reason is that escape routes, such as other splicing isoforms or transcripts from duplicate genes, can partly compensate for the loss of protein abundance caused by the presence of VuORFs. In addition, we cannot exclude the possibility that other regulatory elements dominate translational regulations, and VuORFs actually play a relatively minor role in affecting protein expression level. With their relatively small fitness effects, some VuORFs may have been tolerated in species with a small N_e_. And a small proportion of these VuORFs might subsequently develop novel regulatory functions, or be integrated into the functional proteome through such mechanisms as programmed ribosomal frameshifting or programmed transcriptional realignment [28]. Such VuORFs may eventually become fixed in the population as biologically functional elements. However, the proportion of “functional” VuORFs remains unknown, and is worth further explorations.

## Materials and Methods

### Data Source

Gene annotations, human-mouse-fruit fly gene orthology, and the corresponding transcripts were downloaded from the UCSC (University of California, Santa Cruz) Genome Browser [29] (http://genome.ucsc.edu/). We only retained the known protein-coding transcripts that have an sAUG and an in-frame stop codon to ensure that the transcripts are complete. For comparison, we also retrieved the transcripts and the corresponding information of human, mouse, and fly using the same criteria from the Ensemble website [30] (version 61; http://www.ensembl.org). SuORFs and VuORFs were identified by using an in-house PERL script. We also downloaded the multiple gnomic sequence alignments of 46 vertebrates and of 14 *Drosophila* species from UCSC for estimations of VuAUG conservation. In the AUG context-related analyses, the transcripts that contain only one VuORF (and no other types of uORFs) were selected as the “single-VuORF transcripts” for subsequent analyses. In case of more than one VuORF transcripts encoded by the same gene, one was randomly selected. For the UCSC dataset, 2,951 human (*Homo sapiens*, hg 19), 2,056 mouse (*Mus musculus,* mm9), and 580 fruit fly (*Drosophila melanogaster*, dm3) genes were found to contain VuORF transcript(s). Similarly, for the Ensembl dataset, 3,230 human, 2,226 mouse, and 580 fruit fly genes were analyzed. The numbers of supporting ESTs and cDNAs (Figure S4) were retrieved separately from the UCSC Genome Browser and the Ensembl website using in-house PERL scripts.

According to the scanning model, the translation machinery binds onto the 5′-cap structure of an mRNA and begins scanning from 5′-cap to 3′ end base by base until it encounters the first AUG triplet to initiate the translation process [31], [32]. The translation initiation also depends on the “context” of the AUG triplets (i.e. the composition of nucleotides surrounding the AUG triplets). The context of a uAUG/sAUG was assumed to be optimal (or suboptimal) if the triplet is preceded by a purine (or pyrimidine) at position −3 [26]. The transcripts to be analyzed were then classified into four categories according to the contexts of their uAUGs and sAUGs: (1) OO (Optimal-Optimal) type – both the uAUG and sAUG of the transcript have an optimal context; (2) OS (Optimal-Suboptimal) type – the uAUG context is optimal but the sAUG context is suboptimal; (3) SO (Suboptimal-Optimal) type – the uAUG context is suboptimal but the sAUG context is optimal; (4) SS (Suboptimal-Suboptimal) type – the contexts for both uAUG and sAUG are suboptimal.

### Random Shuffling Experiments

To examine whether VuORFs are disfavored by natural selection, for each species, we generated a theoretical distribution of the proportion of genes that encode at least one VuORF-containing transcript by randomly shuffling the sequences of 5′UTRs of the analyzed transcripts for 1,000 times. We use a PERL subroutine that is based on the Durstenfeld’s shuffle algorithm [33] to generate the shuffled sequences.

For each reshuffling experiment, the entire set of known transcripts of each species were subject to this 5′UTR shuffling, and the proportion of genes that encode at least one VuORF-containing transcript was calculated.

### Analysis of VuAUG Conservation Across Multiple Species

The human-, mouse-, and fruit fly-based UCSC multiple genomic sequence alignments encompass 46, 29, and 14 genomes, respectively. From these genomes, we only retrieved the mammalian genomes (36 genomes in human-based alignments and 22 genomes in mouse-based alignments) and *Drosophila* genomes (11 genomes) for this analysis. We defined the level of human VuAUG conservation as the proportion of non-human mammalian genomes that contain an AUG triplet at exactly the same genomic position as the human VuAUG according to the UCSC multiple alignments. For mouse and fruit fly, the level of VuAUG conservation was also defined in a similar way. The conservation level C(s) was defined as:
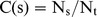
where N_s_ is the number of genomes in which the AUG triplet is conserved; and N_t_ is the total number of compared genomes.

To examine whether the level of VuAUG conservation was significantly higher than expected, we also calculated the conservation level of non-AUG triplets that are composed of A, U, and G nucleotides (i.e. AGU, UAG, UGA, GAU, and GUA) in the examined 5′UTRs. This conservation level was used as a reference “background level”. If the conservation level of VuAUGs was significantly higher than this background level, such VuAUGs were considered as more conserved than expected.

## Supporting Information

Figure S1 **The proportions of genes that encode at least one VuORF transcript in human, mouse, and fruit fly.** The genes here are binned according to the lengths of their 5′UTRs. The pair-wise differences (human-mouse and mouse-fly) in each bin are statistically significant (all *p* values < E−5) except for the human-mouse difference in the leftmost bin (5′UTR length < = 50).
(TIFF)

Figure S2 **The proportions of different types VuORF transcripts in human, mouse, and fruit fly one-to-one orthologous genes.** The table at the bottom shows the statistical significance (by Chi-square test) in pair-wise comparisons between different types of VuORF transcripts. OO: optimal-optimal; OS: optimal-suboptimal; SO: suboptimal-optimal; SS: suboptimal-suboptimal. Statistical significance: *: *p*<0.05; **: *p*<0.01; ***: *p*<0.001; ns: not significant.
(TIFF)

Figure S3 **The proportions of different types VuORF transcripts in human, mouse, and fruit fly.** Note that this figure is based on the Ensembl known transcripts. The table at the bottom shows the statistical significance (by Chi-square test) in pair-wise comparisons between different types of VuORF transcripts. OO: optimal-optimal; OS: optimal-suboptimal; SO: suboptimal-optimal; SS: suboptimal-suboptimal. Statistical significance: *: *p*<0.05; **: *p*<0.01; ***: *p*<0.001; ns: not significant.
(TIFF)

Figure S4 **The numbers of supporting ESTs and cDNAs per gene in human, mouse, and fruit fly according to the UCSC (left panel) and Ensembl data (right panel).**
(TIFF)

Table S1 **The single nucleotide polymorphisms that occur at VuAUGs according to HapMap III.**
(XLSX)
