# Supplementary figures and images for: Selective Constraint on the Upstream Open Reading Frames That Overlap with Coding Sequences in Animals
Source: PLoS One. 2012 Nov 1;7(11):e48413. doi: 10.1371/journal.pone.0048413 (PMC3486843; doi:10.1371/journal.pone.0048413)

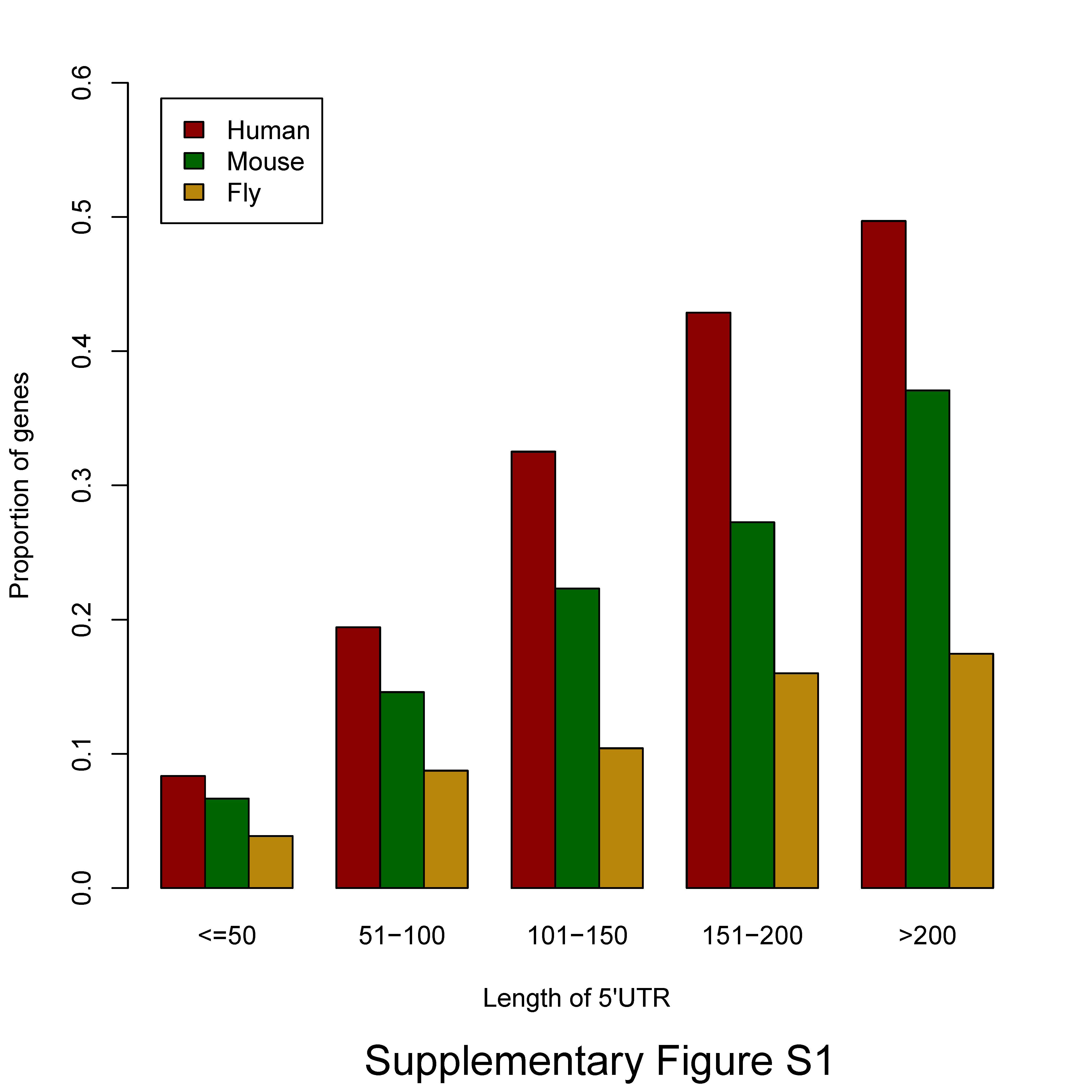

Supplement: Figure S1 — The proportions of genes that encode at least one VuORF transcript in human, mouse, and fruit fly. The genes here are binned according to the lengths of their 5′UTRs. The pair-wise differences (human-mouse and mouse-fly) in each bin are statistically significant (all p values < E−5) except for the human-mouse difference in the leftmost bin (5′UTR length < = 50). (TIFF) [file pone.0048413.s001.tif]

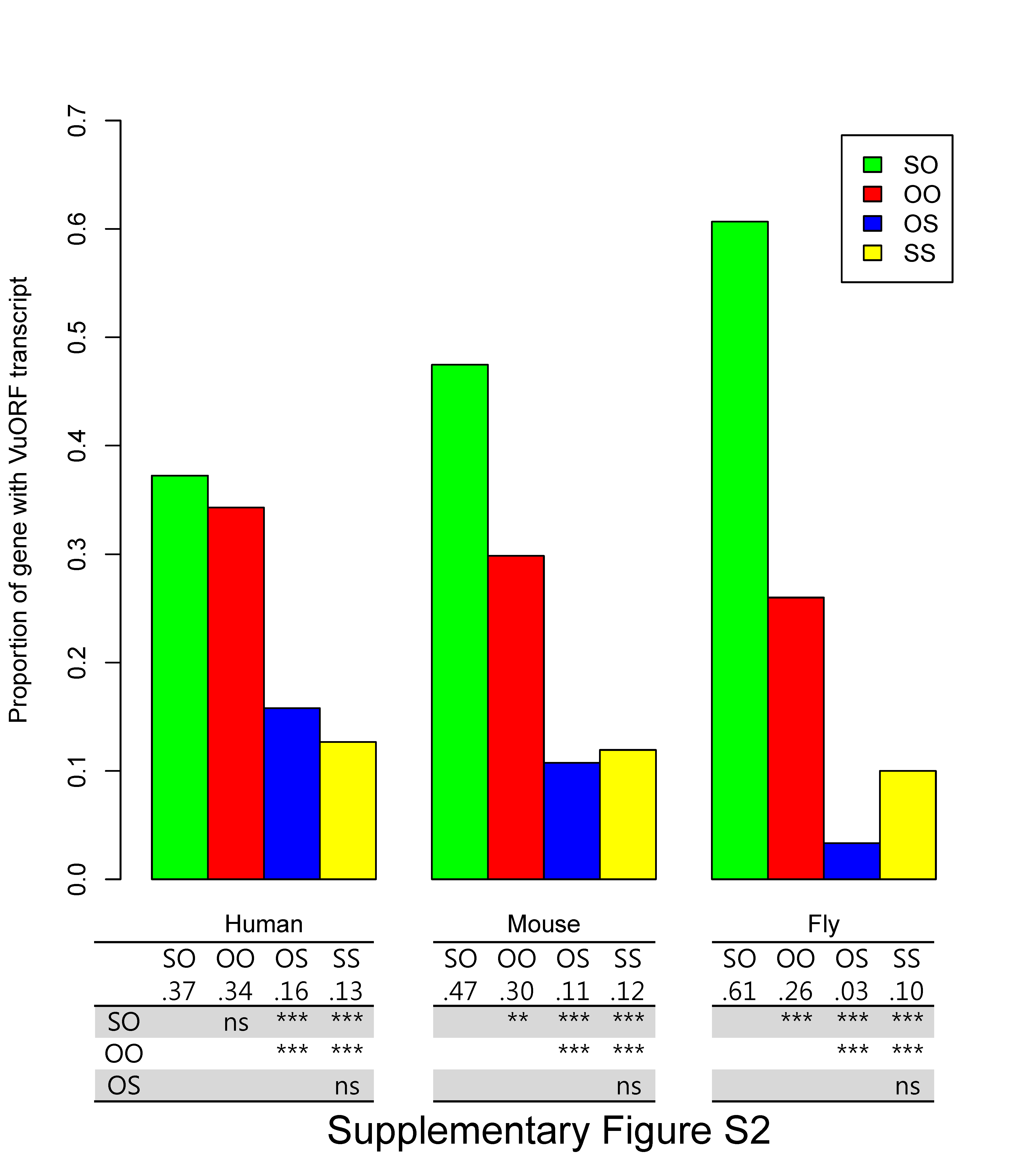

Supplement: Figure S2 — The proportions of different types VuORF transcripts in human, mouse, and fruit fly one-to-one orthologous genes. The table at the bottom shows the statistical significance (by Chi-square test) in pair-wise comparisons between different types of VuORF transcripts. OO: optimal-optimal; OS: optimal-suboptimal; SO: suboptimal-optimal; SS: suboptimal-suboptimal. Statistical significance: *: p<0.05; **: p<0.01; ***: p<0.001; ns: not significant. (TIFF) [file pone.0048413.s002.tif]

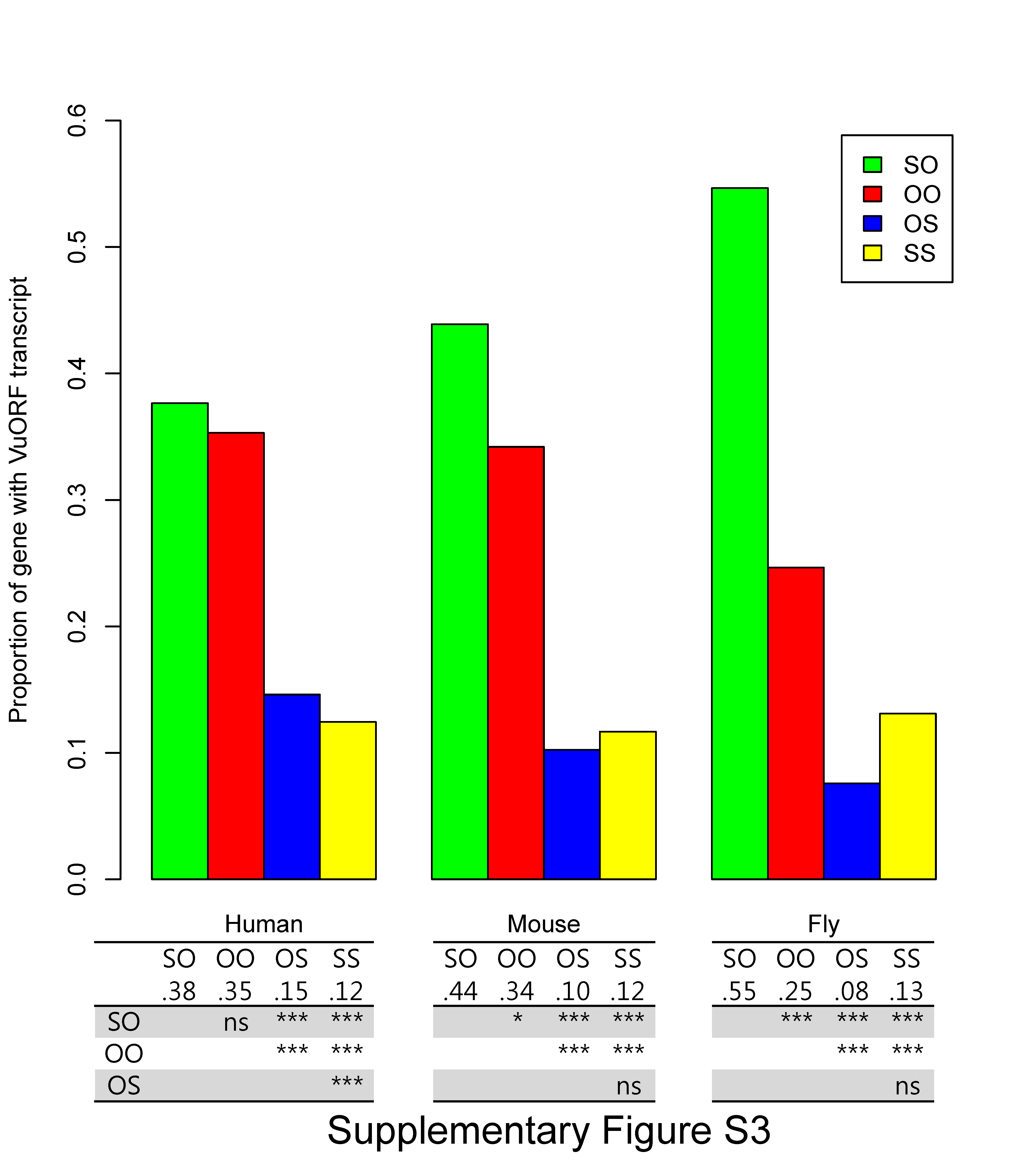

Supplement: Figure S3 — The proportions of different types VuORF transcripts in human, mouse, and fruit fly. Note that this figure is based on the Ensembl known transcripts. The table at the bottom shows the statistical significance (by Chi-square test) in pair-wise comparisons between different types of VuORF transcripts. OO: optimal-optimal; OS: optimal-suboptimal; SO: suboptimal-optimal; SS: suboptimal-suboptimal. Statistical significance: *: p<0.05; **: p<0.01; ***: p<0.001; ns: not significant. (TIFF) [file pone.0048413.s003.tif]
